# SNAIL1 action in tumor cells influences macrophage polarization and metastasis in breast cancer through altered GM-CSF secretion

**DOI:** 10.1038/s41389-018-0042-x

**Published:** 2018-03-29

**Authors:** Audrey Brenot, Brett L. Knolhoff, David G. DeNardo, Gregory D. Longmore

**Affiliations:** 1 ICCE Institute, Washington University, St Louis, MO 63110 USA; 2 Department of Medicine, Washington University, St Louis, MO 63110 USA; 3 Department of Cell Biology and Physiology, Washington University, St Louis, MO 63110 USA

## Abstract

The EMT inducer SNAIL1 regulates breast cancer metastasis and its expression in human primary breast tumor predicts for poor outcomes. During tumor progression SNAIL1 has multiple effects in tumor cells that can impact metastasis. An inflammatory tumor microenvironment also impacts metastasis and recently SNAIL1 has been implicated as modulating the secretion of cytokines that can influence the tumor immune infiltrate. Using a spontaneous genetic model of breast cancer metastasis and syngeneic orthotopic transplant experiments we show that the action of SNAIL1 in primary breast tumor cells is required for breast tumor growth and metastasis. It does so, in part, by regulating production of GM-CSF, IL1α, IL-6, and TNFα by breast cancer cells. The SNAIL1-dependent tumor cell secretome modulates the primary tumor-associated macrophage (TAM) polarization. GM-CSF alone modulates TAM polarization and impacts breast cancer metastasis in vivo. This study highlights another role for breast tumor SNAIL1 in cancer progression to metastasis—modulation of the immune microenvironment of primary breast tumors.

## Introduction

Breast cancer is the most prevalent cancer among women. Despite significant advances in diagnostic modalities and treatments, metastatic spread of breast cancer still results in high mortality rate. Cancer metastasis is a multistep process characterized by local invasion, intravasation, transit through the circulation, extravasation, and survival and proliferation at distant sites. Due to this multistep nature of cancer metastasis there are many cell biological processes that can vary depending upon anatomic localization. One such process, epithelial to mesenchymal transition (EMT), has been implicated as contributing to metastasis at the primary site, during hematogenous spread, and at the metastatic site^1,2^. Importantly EMT exhibits a great deal of plasticity, or reversibility, particularly at the different anatomic locations, or environments, during cancer progression to metastasis. At the primary tumor site, activation of this program in tumor cells is thought to contribute to tumor cell invasion and migration, allowing tumor cells to exit the primary tissue to metastasize^3^.

Several transcription factors act as EMT inducers during normal development and cancer progression to metastasis. SNAIL1, in particular, is a major regulator of early developmental EMT (gastrulation) and genetic deletion of SNAIL1 in breast tumor cells dramatically inhibits metastasis in mouse models of breast cancer^4,5^. The action of SNAIL1 has been implicated in multiple cellular processes including, cell proliferation and survival, cell invasion and migration, and tumor initiating potential^6^. Within breast tumors SNAIL1 is expressed in mammary carcinoma cells as they progress to invasiveness, as well as in cells within the tumor stroma^7^. SNAIL1 protein expression in carcinomas seems to be particularly enhanced in cells at the tumor-stromal interface^7^. In human breast tumors SNAIL1 expression in primary breast cancer cells is associated with higher recurrence, more aggressive tumors, and poorer outcomes^8^.

An inflammatory microenvironment is a well-recognized hallmark of cancer progression^9^. Macrophages, in particular, are observed at the invasive front of the primary breast tumors^10^. Macrophages display phenotypic and functional plasticity, and as such can be divided into two major subsets: classical activation (M1-like) and alternative activation (M2-like)^11^. Although classicaly activated tumor-associated macrophages (TAM) can restrain cancer development, alternatively activated TAM often play a protumorigenic role in that they can promote tumor cell migration and metastasis by influencing immunosuppression, angiogenesis, and ECM deposition and remodeling^10–12^. Indeed, infiltration or enrichment of tumors with TAMs is associated with a poor prognosis in many human tumors^13^.

Whether SNAIL1 can influence the inflammatory microenvironment of tumors to further facilitate metastasis, and if so how, has been addressed in a number of models. SNAIL1 has been shown to regulate inflammatory cytokines and chemokines in several different cell types (macrophages, keratinocytes, melanoma cells, and head and neck cancer cells)^14–19^. In some instances these cytokines have been shown to modulate the immune infiltrates within tumors and tumor size and/or metastasis^16–18^. However, most of these studies used tumor cells that constitutively overexpressed SNAIL1, using vectors that would preclude transcriptional regulation of SNAIL1 in these cells and is a situation that likely does not occur de novo during tumor development and progression. In fact SNAIL1 levels change within tumor cells during tumor progression, and persistent expression of SNAIL1 actually can inhibit metastasis^4^. In addition, all in vivo studies were orthotopic transplants of genetically manipulated tumor cell lines which could induce a different immune infiltrate than spontaneous tumor models. Finally, in addition to inflammatory genes, SNAIL1 regulates expression of genes known to regulate tumor cell migration, adhesion, proliferation, and survival which are all involved in tumor growth and metastasis. Therefore to demonstrate a specific role of SNAIL1 regulated immune infiltrate in tumor metastasis, one needs to evaluate cytokines independent of the other SNAIL1 regulated factors that impact metastases.

In this study we show, in both a spontaneous breast cancer model in which the *Snail1* gene was genetically deleted in tumor cells and an orthotopic syngeneic transplant model comparing SNAIL1-containing with SNAIL1-depleted tumor cells that the presence of SNAIL1 in breast tumor cells regulates secretion of inflammatory mediators that influence the polarization of the TAMs to a tumor promoting phenotype. We then show that select modulation of GM-CSF alone, an inflammatory mediator whose production by tumor cells is controlled by SNAIL1, plays a significant role in TAM polarization at the primary tumor site and cancer progression to metastasis. These results indicate that SNAIL1-mediated production of GM-CSF by tumor cells is another important pathway whereby SNAIL1 regulates breast cancer metastasis.

## Results

### Deletion of SNAIL1 in the breast epithelium reduces breast cancer progression and metastasis to the lung

To dissect the role of SNAIL1 in breast cancer development, progression, and metastasis, we analyzed its role in a spontaneous genetic model of breast cancer metastasis. The *Snail1* gene was deleted in luminal epithelial cells of breast tumors by generating MMTV-Cre, *Snail1*^fl/fl^, MMTV-PyMT mice (SNAIL1 KO, PyMT). Breast tumors that developed in SNAIL1 KO mice exhibited reduced growth, as shown by a decreased tumor burden at 13 weeks (Fig S1A). This delay in tumor formation has been recently observed^5^ and attributed to a reduction in repression of wild type p53, in the absence of SNAIL1, resulting in decreased expansion and activity of the tumor initiating cells. Grossly, SNAIL1 KO tumors appeared round, well demarcated from the surrounding tissues, and less flattened and invasive than WT tumors (Fig S1B, example Fig S1C). Histologic comparison of WT, PyMT, and SNAIL1 KO, PyMT tumors revealed significantly less advanced tumors in the SNAIL1 KO, PyMT mice (Fig S1D with representatives images in Fig S1E). As previously shown^5^, the reduced tumor growth was not due to impaired development of the mammary gland, as the mammary epithelium in 4 week and 5 week old MMTV-Cre, *Snail1*^fl/fl^ females (SNAIL1 KO) was not different than the epithelium of age-matched wild type females (WT) (Fig S1F with an example shown in Fig S1G) as assessed by whole mount staining. Deletion of SNAIL1 also led to reduction of SLUG/SNAIL2 expression indicating that SNAIL1 directly or indirectly regulates levels of SLUG (Fig S2A).

**Fig. 1:**
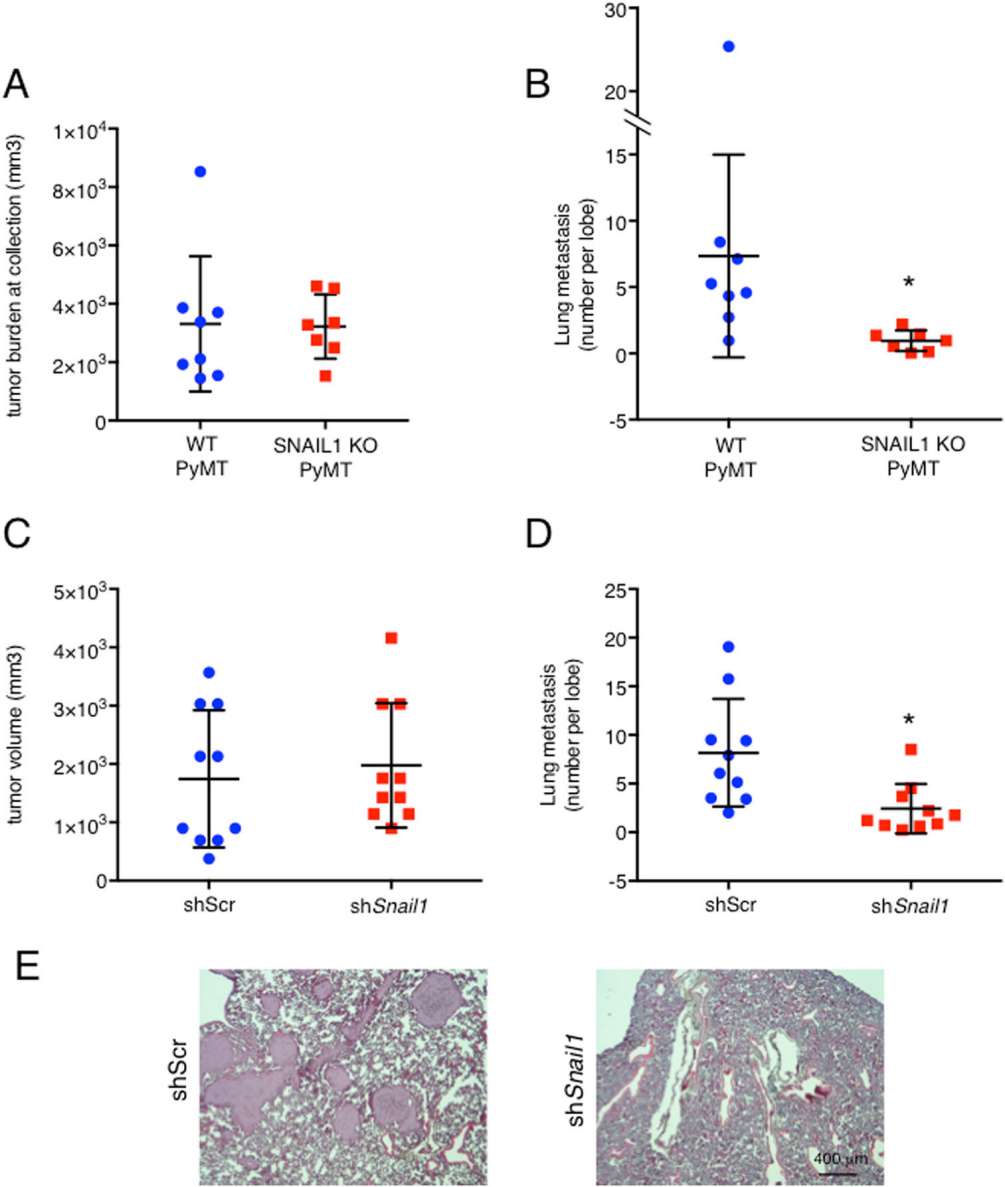
SNAIL1 is required for breast cancer metastasis. **a** Total tumor burden of MMTV-PyMT, *Snail1*^fl/fl^ (WT, PyMT) and MMTV-PyMT, MMTV-Cre, *Snail1*^fl/fl^ (SNAIL1 KO, PyMT) mice, determined by the sum of the volume of all tumors for each mouse at time of collection (when the largest tumor is 10–15 mm in diameter). Seven to eight mice per group. **b** Quantification of lung metastasis when the largest primary tumor (as represented in Fig. 1a) is 10–15 mm. The average number is calculated by counting microscopically visible metastases on each lobe of H&E stained 5 μm sections. Seven to eight mice per group. Tumor volume (**c**) and average number of lung metastasis (**d**) of 4T1-shScr (shScr) and 4T1-sh*Snail1* (sh*Snail1*) mammary xenografts 21 days after transplants. 10 mice per group. **e** Representative images of H&E stained lung sections from mice transplanted with 4T1-shScr (shScr) and 4T1-sh*Snail1* (sh*Snail1*). Scale bar, 400 μm. Data are presented as mean ± SD. *p < 0.05

To assess the role of SNAIL1 in metastasis (independent of its role in primary tumor growth), mice with equivalent primary tumor burden (biggest tumor between 10 and 15 mm) were assessed for lung metastasis. Compared to WT size-matched mice (Fig. 1a), SNAIL1 KO, PyMT mice showed significantly reduced number of lung metastasis (Fig. 1b). To test if this finding was restricted to the MMTV-PyMT spontaneous model, the role of SNAIL1 in a syngeneic orthotopic transplant model was assessed. Equal numbers of mouse metastatic 4T1 cells; either shScr control or SNAIL1-depleted (sh*Snail1*) (Fig S2B) were implanted in the mammary fat pad of syngeneic WT Balb/C female mice. In contrast to the spontaneous tumor model, there was no difference in the primary tumor burden between the two groups of mice (Fig. 1c). This could reflect the fact that SNAIL1 is a suppressor of wild type p53, not mutant p53^5^ and 4T1 cells are p53 null^20^. Despite the absence of difference in primary tumor size, mice implanted with *Snail1*-depleted cells had significantly less lung metastases (Fig. 1d, e). Although the number of lung metastases were reduced when *Snail1* was either depleted in syngeneic orthotopic transplant or deleted in spontaneous tumor model, respectively, the size of individual metastases was not different between WT and sh*Snail1* or SNAIL1 KO mice (Fig S2C and Fig S2D, respectively).

In sum, these data indicated that SNAIL1 played a critical role in the growth of the primary tumor in the context of wild type p53, but also that tumor cell SNAIL1 influenced breast cancer metastasis in both a spontaneous MMTV-PyMT p53 wild type genetic model and a p53 null syngeneic orthotopic transplant model, suggesting a p53-independent role of Snail1 in metastastic progression.

### Tumor cell SNAIL1 modulates TAM polarization in primary tumors

Inflammation within tumors can dramatically impact tumor progression both positively and negatively. SNAIL1 is a transcriptional regulator that directly and indirectly affects the transcription of many genes, including inflammatory modulators^15,17,18^. Therefore, we asked whether the action of tumor cell-intrinsic SNAIL1 affected tumor growth and metastasis by changing the inflammatory tumor microenvironment. We characterized the immune infiltrates present in SNAIL1 expressing and SNAIL1 deleted (SNAIL1 KO or sh*Snail1*) primary tumors, in the spontaneous MMTV-PyMT and syngeneic orthotopic transplant model.

**Fig. 2:**
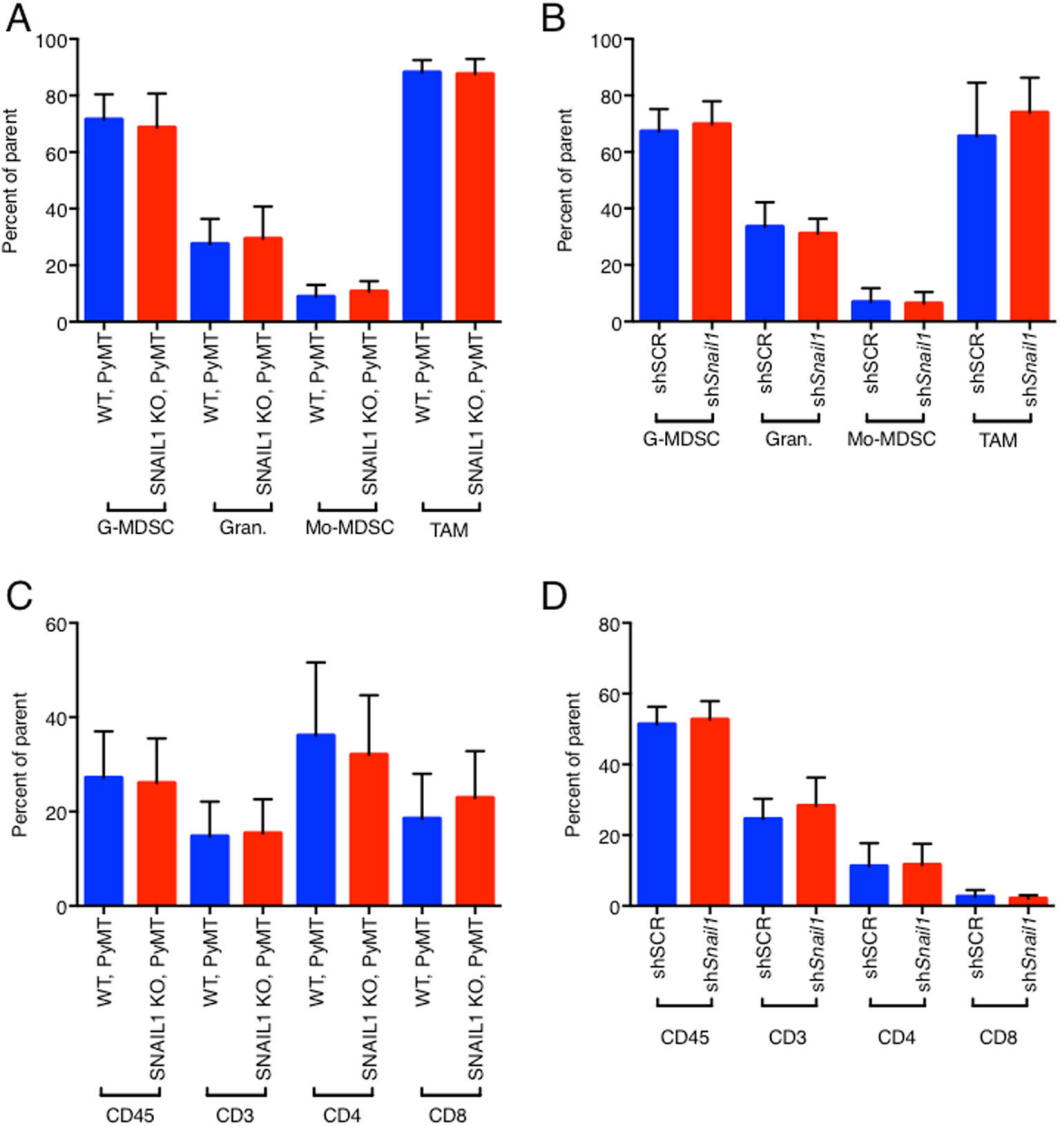
Immune infiltrates in tumors. Immunophenotyping of tumors from MMTV-PyMT, *Snail1*^fl/fl^ (WT, PyMT) and MMTV-PyMT, MMTV-Cre, *Snail1*^fl/fl^ (SNAIL1 KO, PyMT) mice (**a**, **c**) or 4T1-shScr (shScr) and 4T1-sh*Snail1* (sh*Snail1*) transplanted mice (**b**, **d**). The frequency of G-MDSCs (CD11b^+^Ly6G^+^Ly6C^+^MHCII^Low^), Granulocytes (Gran.) (CD11b^+^Ly6G^+^Ly6C^-^MHCII^Hi^), Mo-MDSC (CD11b^+^Ly6G^-^Ly6C^+^), TAM (CD11b^+^Ly6G−Ly6C−F4/80^+^MHCII^+^) (**a**, **b**) and leukocytes (CD45^+^), T cells (CD3^+^), T helper cells (CD3^+^CD4^+^) and cytotoxic T cells (CD3^+^CD8^+^) (**c** and **d**) is depicted as the mean percentage of parent cells (as described in more details in Material and Methods). All graphs depict mean values +/− SD, *n* = 10 mice per group for orthotopic transplants and 17 to 20 tumors (7 mice each) per group for the transgenic model, in all graphs, *p* > 0.05 by unpaired *t*-test

**Fig. 3:**
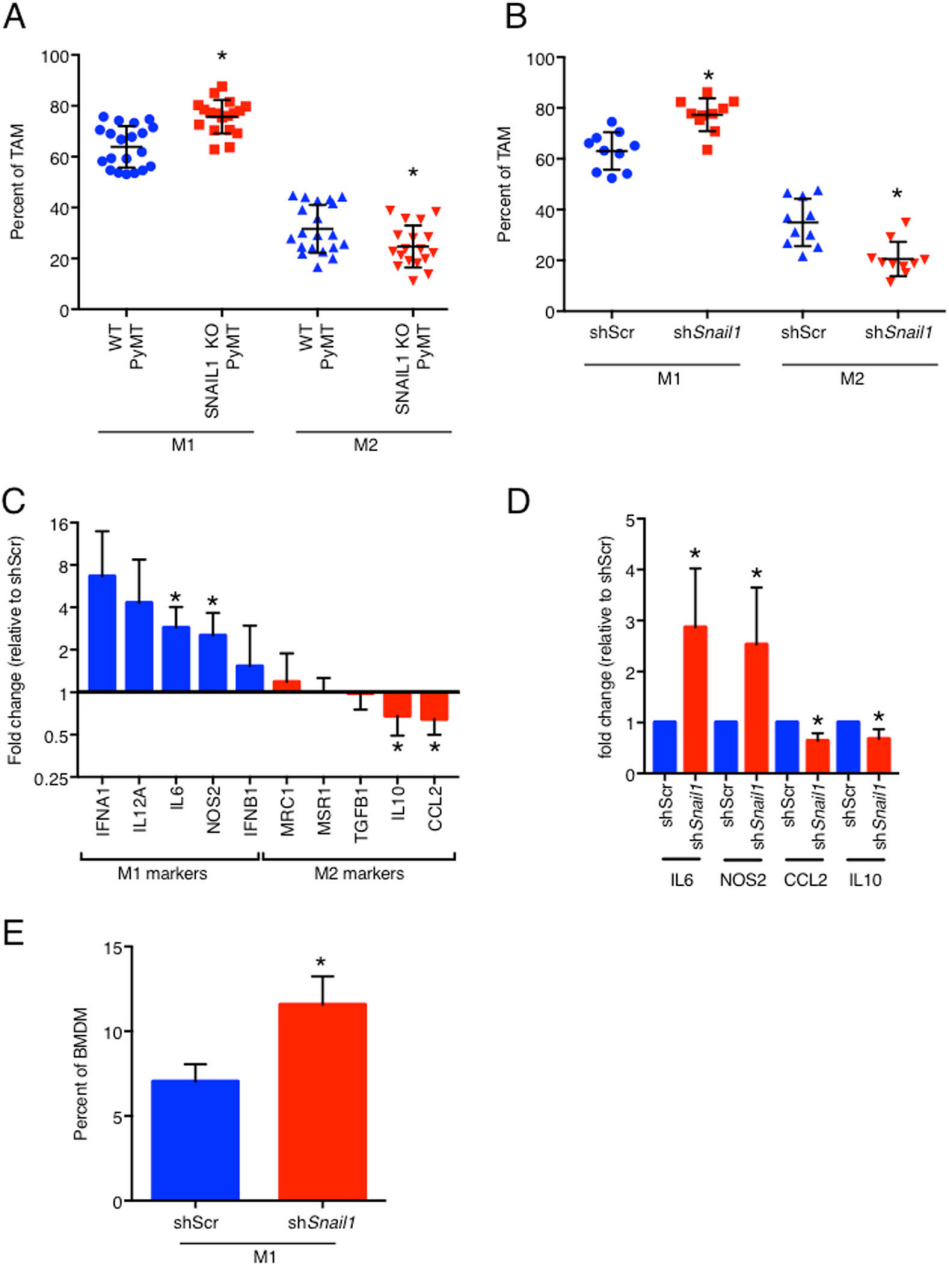
SNAIL1 modulates macrophage polarization in vivo and in vitro. **a** Quantification of tumor infiltrated M1-like and M2-like macrophages in MMTV-PyMT, *Snail1*^fl/fl^ (WT, PyMT) and MMTV-PyMT, MMTV-Cre, *Snail1*^fl/fl^ (SNAIL1 KO, PyMT) mice when the largest primary tumor reaches 10–15 mm expressed as a percent of total TAM. 18–20 tumors per group (seven mice each). **b** Quantification of tumor infiltrated M1-like and M2-like macrophages in 4T1-shScr (shScr) and 4T1-sh*Snail1* (sh*Snail1*) mice 21 days post transplant expressed as a percent of total TAM. Ten tumors per group. **c**, **d** qPCR analysis on TAMs sorted from tumors 14 days post transplant expressed as the fold change of gene expression in 4T1-sh*Snail1* relative to 4T1-shScr. Four mice per group. All the genes analyzed are grouped as M1-like and M2-like molecules (**c**) and the significantly regulated genes are represented in (**d**). **e** In vitro M1-like polarization of bone marrow derived macrophages (BMDM) by conditioned media from 4T1-shScr (shScr) or 4T1-sh*Snail1* (sh*Snail1*) expressed as a percent of total BMDM. Representative results of two biological replicates with three technical replicates. Data are presented as mean ± SD. *p < 0.05

Immune cells important for the establishment of an immunosuppressive tumor microenvironment include tumor-associated macrophages (TAM), monocytic myeloid-derived suppressor cells (Mo-MDSC) and granulocytic MDSCs (G-MDSC). These leukocytes regulate the adaptive lymphocytic response to tumors, and as such, can influence tumor growth and metastasis^21,22^. In the spontaneous tumor model, most myeloid cell populations (CD45^+^ CD11b^+^ Ly6G^+^ Ly6C^+^ MHCII^low^/G-MDSC, CD45^+^ CD11b^+^ Ly6G^+^ Ly6C^−^ MHCII^high^/Mature Granulocytes, CD45^+^ CD11b^+^ Ly6G^−^ Ly6C^+^/Mo-MDSC as defined in^23^) and total number of TAMs (CD45^+^ CD11b^+^ Ly6G^−^ Ly6C^−^ F4/80^+^ MHCII^+^) present in primary MMTV-PyMT tumors+/− *Snail1* were not significantly different (Fig. 2a). Similarly, in the syngeneic orthotopic transplant model there were no changes in myeloid cell numbers in sh*Snail1* tumors (Fig. 2b). There was no significant difference in total T cells (CD3^+^), T helper cells (CD3^+^CD4^+^) or cytotoxic T cells (CD3^+^CD8^+^) in SNAIL1 KO or sh*Snail1* primary tumors in both models (Fig. 2c, d). There was also no significant change in the percent of activated CD4 T cells (Fig S2E) or activated CD8 T cells (Fig S2F). However, a higher percent of TAMs were polarized towards an M1-like phenotype (CD45^+^ CD11b^+^ Ly6G^−^ Ly6C^−^ F4/80^+^ MHCII^+^ CD206^−^) in SNAIL1 KO tumors, while the percent of M2-like macrophages (CD45^+^ CD11b^+^ Ly6G^−^ Ly6C^−^ F4/80^+^ MHCII^+^ CD206^+^) was decreased (Fig. 3a). In the syngeneic orthotopic transplant model the same increase in M1-like and decrease in M2-like macrophages was observed in the tumor infiltrating leukocyte population of *Snail1*-depleted tumors (Fig. 3b).

Classically activated macrophages (M1-like TAM) typically express proinflammatory cytokines like inducible nitric oxide synthase 2 (NOS2) and MHC II molecules and can restrain cancer development and metastasis. Alternatively activated macrophages (M2-like TAM) have a decreased expression of the above markers, increased expression of the mannose receptor (CD206) and often play a protumorigenic role^24,25^. To better characterize the effects of depleting SNAIL1 from the tumor cells on the TAM population in the primary tumor, we analyzed gene expression of molecules known to be differentialy expressed in these two subsets^23,26–28^. TAMs were FACS-sorted from 4T1-shScr and 4T1-sh*Snail1* tumors 14 days after transplants and gene expression compared. TAMs from SNAIL1 depleted tumors displayed increased expression of M1-like/anti-tumor genes like Ifna1, Il12a, Il6, Nos2, and Ifnb1. In contrast M2-like/immunosuppressive genes like Mrc1, Msr1, Tgfb1, Il10, and Ccl2 displayed similar or reduced expression (Fig. 3c). Amongst these genes, the expression of the M1-like genes Il6 and Nos2 was significantly increased while the expression of the M2-like genes Ccl2 and Il10 was significantly decreased when SNAIL1 was depleted (Fig. 3d) demonstrating that depletion of SNAIL1 in the tumors cells led to an increase in functional M1-like or classicaly activated macrophages.

To determine how the action of SNAIL1 in tumor cells affected TAM polarization we asked whether the presence or absence of SNAIL1 altered the tumor cell secretome, and thus, regulated TAM polarization through a paracrine mechanism. Bone marrow derived macrophages were cultured in the presence of conditioned media from sh*Snail1*-depleted 4T1 cells or parental 4T1 cells. Under these conditions there was a greater proportion of macrophages polarized towards an M1-like phenotype in the presence of conditioned media from 4T1 cells depleted of SNAIL1 compared to their polarization in the presence of conditioned media from SNAIL1-containing 4T1 cells (Fig. 3e). To confirm that this finding was not limited to 4T1 cells, SNAIL1 expression was depleted in a cell line derived from MMTV-PyMT tumor cells (PMT-shScr and PMT-sh*Snail1*). Similar to the findings with the 4T1 cells, conditioned media from the SNAIL1-depleted PyMT cells induced polarization of a greater number of macrophages towards an M1-like phenotype (Fig S3A).

### SNAIL1 regulates cytokine/chemokine production in breast cancer cells

**Fig. 4:**
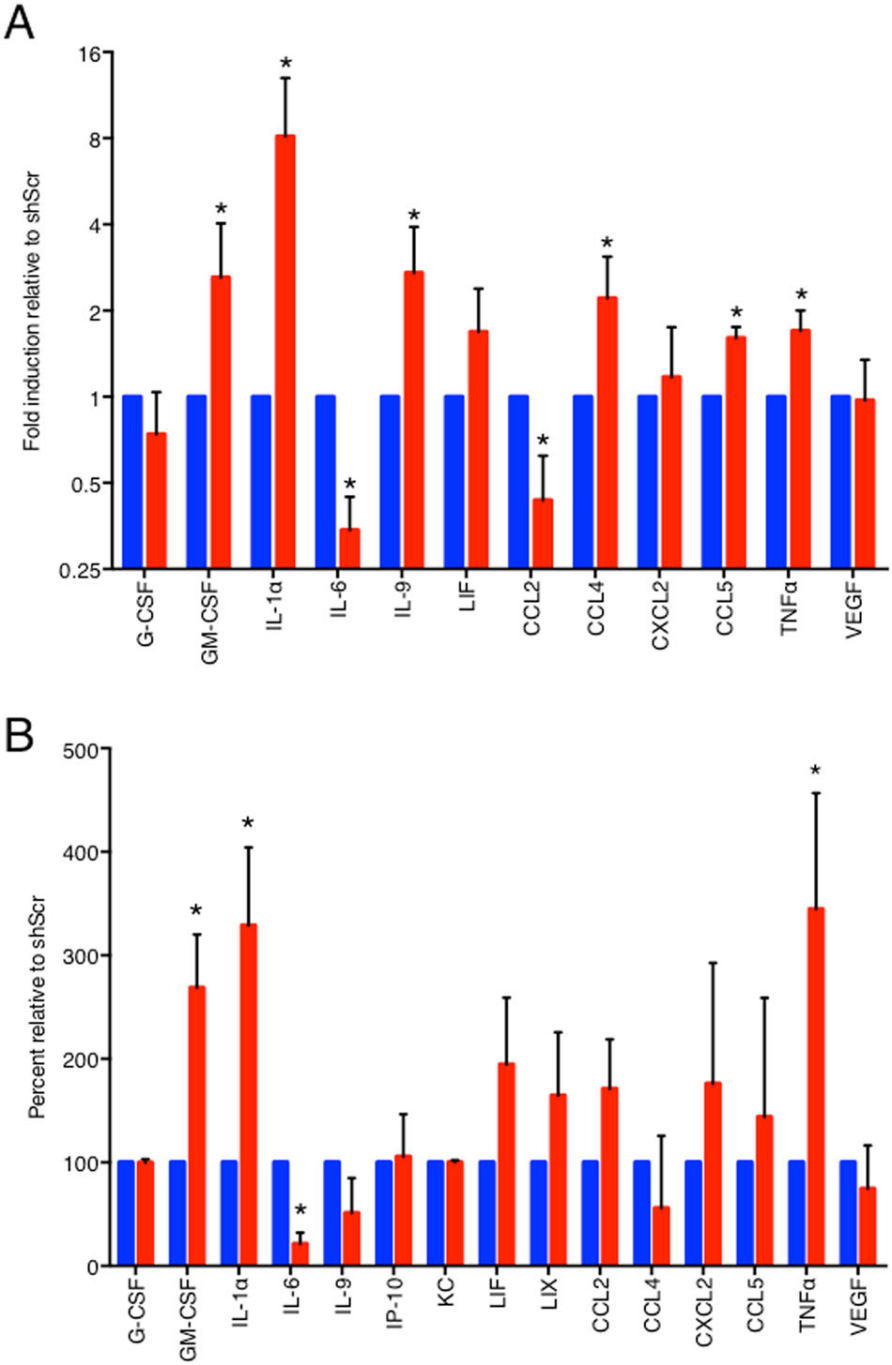
SNAIL1 regulates cytokine/chemokine production in breast cancer cells. **a** qPCR analysis of mRNA isolated from 4T1-shScr (blue) and 4T1-sh*Snail1* cells (red) with 2 ng/ml TGFβ. Each gene was quantified from two biological replicates with three technical replicates each and normalized to 4T1-shScr. **b** Relative levels of cytokines and chemokines in conditioned media from 4T1-sh*Snail1* cells (red) compared to 4T1-shScr cells (blue). Cells were grown with 2 ng/ml TGFβ and protein levels were quantified in filtered supernatants by Multiplex ELISA. Each protein was quantified from three biological replicates and normalized to the protein levels in 4T1-shScr. Data are presented as mean ± SD. *p < 0.05

Since conditioned media from 4T1 breast tumor cells depleted of SNAIL1 was able to recapitulate macrophage polarization observed in vivo in primary breast tumors lacking *Snail1*, we analyzed and contrasted the cytokines and chemokines expressed by parental 4T1 and SNAIL1-depleted 4T1 breast tumor cells. Analysis of mRNA levels by qRT-PCR revealed increased levels of GM-CSF, IL-1α, IL-9, CCL4, CCL5, and TNFα and decreased levels of IL-6 and CCL2 mRNA in SNAIL1-depleted cells (Fig. 4a). Using a multiplex ELISA, we determined the level of 31 cytokines and chemokines. Depletion of SNAIL1 led to significantly increased secretion of GM-CSF (also called CSF-2), IL1α, and, TNFα as well as significantly decreased secretion of IL-6 (Fig. 4b). However, by ELISA analysis, the cytokines and chemokines IL-9, CCL2, CCL4, and CCL5 were not secreted at significantly different levels (Fig. 4b). We decided to focus on GM-CSF as it is known to regulate macrophage polarization^29,30^.

### GM-CSF recapitulates the macrophage polarization and metastasis of *Snail1*-deleted tumors

To test whether GM-CSF alone could recapitulate the various in vitro and in vivo tumor phenotypes observed in mice with *Snail1*-deleted tumors, we employed multiple approaches.

**Fig. 5:**
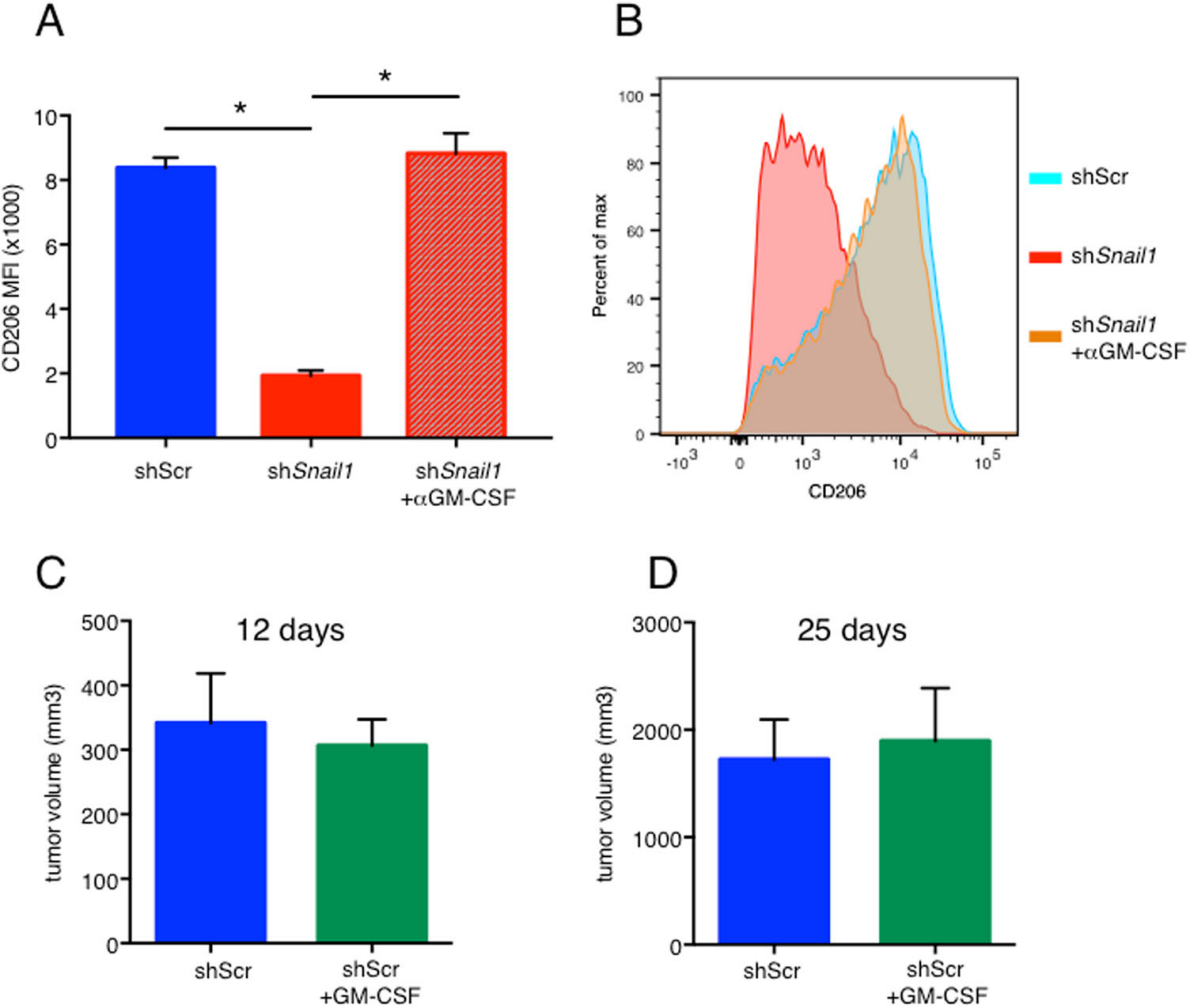
GM-CSF rescues the macrophage polarization in vitro. **a** In vitro polarization of BMDM by conditioned media from 4T1-shScr (shScr), 4T1-sh*Snail1* (sh*Snail1*) and 4T1-sh*Snail1* in the presence of anti GM-CSF antibody (sh*Snail1* + αGM-CSF) expressed as the CD206 Mean Fluorescence Intensity (MFI) of TAM with representative image in **b**. Representative results from two biological replicates with technical triplicates. Average tumor volume of mice orthotopically transplanted with 4T1-shScr (shScr) cells and 4T1-shScr cells with injections of GM-CSF (shScr + GM-CSF) 12 days (**c**) and 25 days (**d**) post orthotopic transplant. Data are presented as mean ± SD. *p < 0.05

First, a neutralizing anti-GM-CSF antibody was added to conditioned media from sh*Snail1* 4T1 cells and macrophage polarization capacity of this GM-CSF-neutralized conditioned media assessed with WT BMDM. Neutralization of GM-CSF resulted in a macrophage polarization profile seen when culture supernatant from control shScr 4T1 cells (no anti-GM-CSF) was added to BMDM; more towards an M2-like phenotype, as shown by an increase in CD206 mean fluorescence intensity, a marker of M2-like macrophages (Fig. 5a, representative image Fig. 5b).

**Fig. 6:**
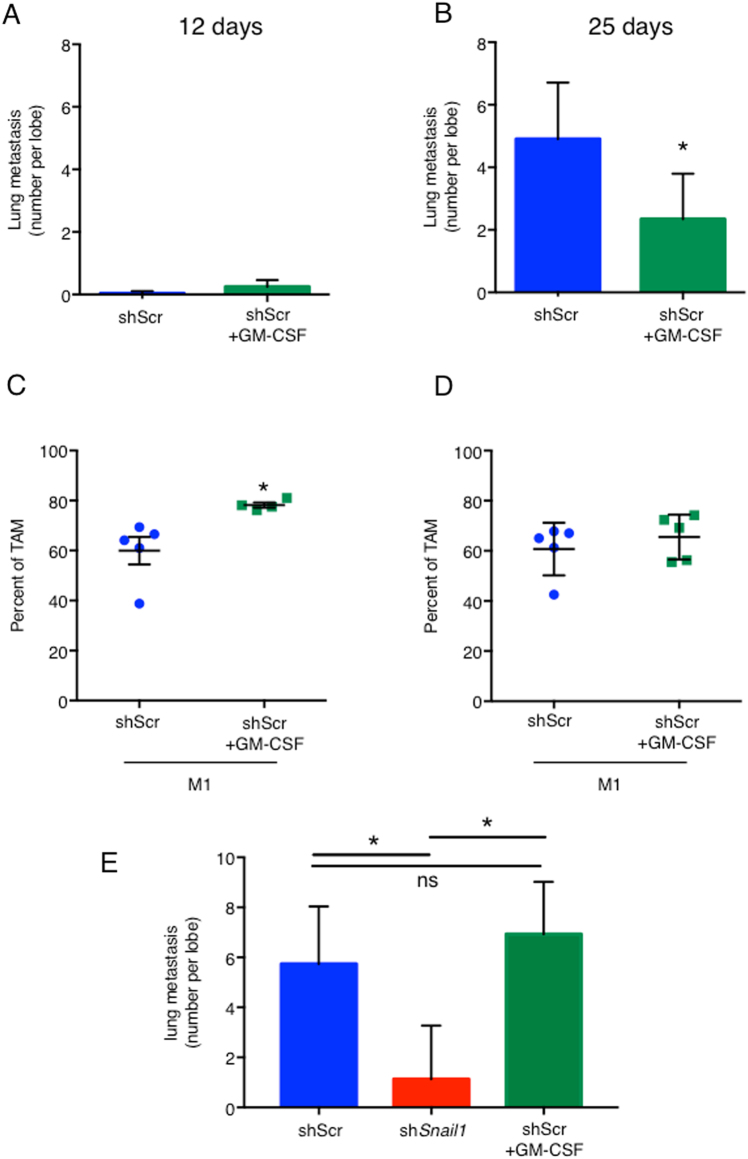
Rescue of lung metastasis and macrophage polarization in vivo with the addition of GM-CSF. Average number of lung metastasis of mice orthotopically transplanted with 4T1-shScr (shScr) cells and 4T1-shScr cells with injections of GM-CSF (shScr + GM-CSF) 12 days (**a**) and 25 days (**b**) post orthotopic transplant. Four to five mice per group. Quantification of tumor infiltrated M1-like macrophages in tumors from 4T1-shScr (shScr) transplants and 4T1-shScr with injections of GM-CSF (shScr + GM-CSF) 12 days (**c**) and 25 days (**d**) post transplant. Four to five mice per group. **e** Average number of lung metastasis of mice intravenously injected with 4T1-shScr (shScr) cells, 4T1-sh*Snail1* (sh*Snail1*) and 4T1-shScr cells with injections of GM-CSF (shScr + GM-CSF) 7 days post IV injection. Data are presented as mean ± SD. *p < 0.05 and ns is not significant (*p* > 0.05)

In a second approach, mice were treated for 7 days with recombinant GM-CSF or saline solution, then mammary glands transplanted with 4T1-shScr tumor cells and GM-CSF or saline solution treatment continued for the duration of the experiment. Treatment with GM-CSF did not affect primary tumor growth at 12 days (Fig. 5c) or 25 days (Fig. 5d). Lung metastases at 12 days were very rare in this model and their number was not affected by treatment with GM-CSF (Fig. 6a). But at 25 days, the metastatic burden in the lung was significantly reduced in mice treated with GM-CSF (Fig. 6b). Treatment with GM-CSF also increased the percentage of M1-like macrophages in primary tumors at 12 days post transplant (Fig. 6c) while it was not significantly different at 25 days (Fig. 6d). The decrease in number of lung metastasis at 25 days in the presence of GM-CSF (Fig. 6b) could, in part, result from the increase in M1-like polarization at 12 days (Fig. 6c). The fact that there was no difference in macrophage polarization in the presence of GM-CSF at 25 days (Fig. 6d) is perhaps not surprising since the tumors at that stage are necrotic.

The number of other myeloid populations and T cell populations were not significantly changed at either time points (Fig S3B-E). When cells were directly introduced into the circulation through IV injection, depletion of SNAIL1 resulted in a significant reduction in lung metastasis, but treatment with GM-CSF did not significantly reduce the number of metastasis when WT cells were injected (Fig. 6e). This indicated that, while Snail1 was required for metastatic growth in the lungs in the tail vein assay, the GM-CSF mediated effect of Snail1 on lung metastasis was most likely a local effect within the primary tumor and not a systemic effect.

The extent of altered M1-like TAM polarization observed with GM-CSF treatment in the orthotopic model (Fig. 6c) was equivalent to that observed in mice transplanted with *shSnail1*-depleted 4T1 cells (Fig. 3b), indicating that GM-CSF was likely a major regulator of M1-like polarization in breast tumors lacking SNAIL1.

Collectively our data suggest that GM-CSF production in SNAIL1-depleted breast tumor cells, plays a significant role in inhibiting breast tumor metastasis to the lung by influencing the polarization of infiltrating macrophages within the primary tumor.

## Discussion

The action of SNAIL1 in breast tumor cells has been shown, in mouse models, to be a key regulator of metastasis^4,5,31,32^. The presence of SNAIL1 in human primary breast tumors predicts for poor clinical outcomes with increased tumor grade, nodal metastasis, and tumor recurrence^5,8,33^. SNAIL1 contributes to these phenotypes through various tumor cell functions including tumor cell invasion and migration^34,35^, tumor cell survival, and proliferation^36^, the maintenance of tumor initiating cells^5,6^, resistance to therapy^6,37^, and recurrence^8^. Recent studies indicate that SNAIL1 is also involved in the regulation of the immune compartment, highlighting another way SNAIL1 is a critical player in cancer progression and metastasis^14,15,17,18^. However, the mechanism by which SNAIL1 regulates the immune compartment remains controversial and conclusions from these studies are limited by constitutive overexpression of *Snail1* in tumor cells, inducing levels of SNAIL1 not present during tumorigenesis nor is the overexpressed SNAIL1 transcriptionally regulated. In fact, endogenous SNAIL1 expression during breast cancer development, progression, and metastasis fluctuates and these changes are critical for efficient metastasis^4^. In the current study, we show a direct in vivo link between SNAIL1 expression in tumor cells and modulation of the immune infiltrate within the primary tumor through GM-CSF secretion that subsequently impacts metastasis to distant sites.

A recent study showed that tumors derived from carcinoma cells expressing low levels of SNAIL1 (Snail^lo^) elicited a strong CD8^+^ T cells and M1 macrophage response while tumors derived from carcinoma cells expressing higher levels of SNAIL1 (Snail^hi^) induced a strong regulatory T cell and M2 macrophage response^38^. The association of lower levels of SNAIL1 with an M1-like response is consistent with our findings. This study did not elucidate the molecular mechanism by which SNAIL1 induced immunosuppression occurs, however.

SNAIL1 has been shown to regulate cytokine and chemokine production in several different cells (macrophages, keratinocytes, melanoma cells), although again most of these studies utilized constitutive overexpression of *Snail1*^14,15,18^. Triple Negative Breast Cancer cell lines (classified as Mesenchymal-type) and breast cancer cell lines that have undergone EMT express higher levels of GM-CSF and activate macrophages to an M2-like phenotype, suggesting that GM-CSF is necessary to induce M2-like polarization of macrophages^16^, and thus, that GM-CSF is pro-tumorigenic. This is perhaps surprising since GM-CSF has been shown to elicit powerful immune responses, is often used as an adjuvant to cancer vaccines^39^ and overexpressing GM-CSF can inhibit tumor growth and metastasis^40^. This is also in contradiction with our findings since we showed in two mouse model of breast cancer metastasis, that GM-CSF expression by tumor cells is repressed by the presence of SNAIL1 and in vivo GM-CSF treatment has an anti-metastatic effect. Furthermore, GM-CSF receptor signaling has been shown to fine tune the molecular profile of M1-like macrophages, although it does not seem to regulate monocyte recruitment and differentiation^26^. These apparent conflicting roles for GM-CSF can be due, at least in part, to differences in GM-CSF dose, influence of other stimulating factors or nature of the existing inflammatory response^41,42^.

In this study, we showed that SNAIL1 regulates expression of cytokines in primary breast tumor cells including IL-1α, IL-6, TNFα, and GM-CSF. SNAIL1 represses the production of pro-inflammatory cytokines IL-1α and TNFα and inhibits production of GM-CSF, a known stimuli of M1-like macrophages leading to a suppression of the pro-inflammatory response elicited by the tumor cells. Different studies have shown that SNAIL1 in tumor cells can regulate different sets of cytokines and chemokines. SNAIL1 activates expression of CCL2, CCL5, IL-6, TNFα, and IL-8 in head and neck cancer cells and overexpression of SNAIL1 in 4T1 cells increased infiltration of M2-like macrophages and promoted metastasis in a breast cancer orthotopic model^17^. We also observed regulation of CCL2, CCL5, IL-6, and TNFα mRNA levels by SNAIL1 but this did not translate to a significant difference in secreted proteins with the exception of IL-6 and TNFα.

Our data indicate that when SNAIL1 expression is activated in tumor cells, they produce less pro-inflammatory cytokines IL1α and TNFα and M1-like stimulating GM-CSF. This leads to a reduction in the pro-inflammatory tumor response, that appears to positively influence metastasis.

B and T lymphocytes also exert pro-tumor activity indirectly by regulating the activity of myeloid cells including macrophages, monocytes and mast cells, resulting in resistance to endocrine therapies and increased metastasis^43^. We did not see any significant difference in the number of total T cells, T helper cells or cytotoxic T cells or in their activation levels suggesting that the action of SNAIL1 in tumor cells has little impact upon recruitment of these immune populations. Our data, however, does not exclude the possibility that regulatory T cells or dendritic cells are affected as it has been observed when overexpressing SNAIL1 in melanoma cells^18^.

In conclusion, we show that the action of SNAIL1 in breast tumor cells modulates the immune microenvironment within breast tumors by regulating expression of cytokines including GM-CSF. In breast cancer, SNAIL1 direct or indirect regulation of GM-CSF appears to have a significant role in the polarization of TAMs and metastases.

## Materials and methods

### Animal studies

BALB/C and FVB mice were purchased from Charles River. Conditional SNAIL1 knock out mice (SNAIL1 KO) were generated by crossing *Snail1*^fl/fl^ mice^44^ with MMTV-Cre mice (Tg(MMTV-cre)4Mam). Inguinal mammary glands number 4 from 4 to 5 week old females were retrieved, Carmine Alum (Sigma-Aldrich) stained and the ductal-invaded area was calculated using ImageJ software. The mammary outgrowth was determined as the area of epithelium growth (as demarcated) divided by the total area of the mammary fat pad. Conditional mice were crossed to MMTV-PyMT mice (SNAIL1 KO, PyMT) to generate mammary tumors. The tumor burden was calculated as the total of the tumor volumes using the equation V = 0.52 × length × (width)^2^. Tumor grading was made blindly as round or invasive as the examples shown in the supplemental figures. For mammary orthotopic transplants, 10^6^ cells were injected in the right inguinal fat pad number 4. For intravenous (IV) injections, 5 × 10^5^ cells were injected in saline solution in the mouse tail vein. No randomization was used as mice were injected with either shScr or sh*Snail1* cells. Sample size calculation for equal variance *t*-test showed the sample size needed was seven per group to achieve the statistical power of 0.9. All procedures involving animals and their care were performed in accordance with the guidelines of the American Association for Accreditation for Laboratory Animal Care and the U.S. Public Health Service Policy on Human Care and Use of Laboratory Animals. All animal studies were also approved and supervised by the Washington University Institutional Animal Care and Use Committee in accordance with the Animal Welfare Act, the Guide for the Care and Use of Laboratory Animals and NIH guidelines (Protocol 20150145).

### Cells, viral production, and transduction

4T1 cells were purchased from ATCC by our laboratory in 2014. Cells were cloned and a high SNAIL1 expressing clone was selected. The PMT cell line was derived from an MMTV-PyMT mammary tumor, immortalized through at least ten passages and FACS sorted for the CD140a (PDGFR, eBioscience Clone APA5) negative population. Viral production was carried out using TransIT-LT1 (Mirus Bio) mediated transfection of HEK293T cells. Virus was concentrated by ultracentrifugation, and added to the cells with Polybrene. 4T1 cells (ATCC) or PMT cells were transduced with GIPZ-SCR or GIPZ-Snai1 (Thermo Scientific, 3 shRNA tested, most efficient knock down selected for subsequent studies) for shRNA knock-down of SNAIL1. Stably transduced cells were selected in puromycin for at least 5 days. Knock-down of SNAIL1 was confirmed by Western Blot. Cells were not passaged more than 15 times. Cells were tested for Mycoplasma by PCR amplification using primers Myco + (5′-GGG AGC AAA CAG GAT TAG ATA CCC T-3′) and Myco− (5′-TGC ACC ATC TGT CAC TCT GTT AAC CTC-3′) every 6 months and treated for a minimum of 2 weeks with Plasmocin (InvivoGen) if the Mycoplasma PCR was positive, until the PCR was negative.

### Western blotting

Cells were lysed in RIPA Buffer plus protease inhibitors (Sigma-Aldrich). Protein concentration was measured using the Bradford Reagent (Biorad). Lysates were subjected to SDS-PAGE, transferred to PVDF membranes, blocked in 5% milk, incubated with primary antibody overnight, secondary antibody for 2 h and visualized using the detection kits SuperSignal WestPico and/or SuperSignal West Femto Chemiluminescent Substrates (Thermo Fisher Scientific). Exposures were acquired using a ChemiDoc Imager (BioRad). Antibodies used include β-actin (A1978, Sigma Aldrich), Mouse SNAIL1 (L70G2, Cell Signaling), SLUG/SNAIL2 (C19G7, Cell Signaling) and HRP anti-Mouse IgG (Cell Signaling).

### Analysis of lung metastasis

Lungs and tumors were fixed overnight in 10% Neutral Buffered Formalin, washed in PBS, 30% ethanol, 50% ethanol, and 70% ethanol then processed and embedded in paraffin blocks. Tissue sections (5–6 μm) were stained with H&E. Microscopically visible metastasis were counted by a blinded investigator from three sections taken 30 μm apart and reported as the average number of metastases per lung lobe. Metastasis size was quantified by ImageJ and expressed as number of pixels.

### Flow cytometry analysis of tumor infiltrated immune cells

For flow cytometry, all mice were perfused with Heparin in PBS, mammary tumors were mechanically dissociated with scissors and the tissue was digested in culture medium (DMEM) with 2 mg/ml Collagenase A (Roche) and 2 μg/ml DNAse (Sigma Aldrich) at 37 °C for 30 min with agitation. Digestion mixtures were quenched by adding 10% FBS and samples were filtered through a 40 μm cell strainer (Falcon). Cells were stained for 20–30 min in the dark on ice with the conjugated antibodies as follows (eBioscience unless otherwise noted) following manufacturer’s recommended concentrations: Myeloid panel: CD45 (30-F11), CD11b (M1/70), MHCII (M5/114.15.2), Ly6C (HK1.4), Ly6G (1A8), F4/80 (BM8), CD206 (19.2, BioRad) and T cell panel: CD45 (30-F11), CD3ε (145-2C11), CD4 (L3T4), CD8α (53–6.7), CD62L (MEL-14), and CD44 (IM7). Cells were then fixed with BD Cytofix (BD Biosciences) and flow cytometry was performed on a BD LSRII flow cytometer (BD Bioscience) and analyzed using FlowJo software (TreeStar). Gating strategy was performed as described in^23^ and the different populations defined as follow: G-MDSC (CD45^+^ CD11b^+^ Ly6G^+^ Ly6C^+^ MHCII^low^), Mo-MDSC (CD45^+^ CD11b^+^ Ly6G^−^ Ly6C^+^), Mature Granulocytes (Gran.) (CD45^+^ CD11b^+^ Ly6G^+^ Ly6C^−^ MHCII^high^), TAMs (CD45^+^ CD11b^+^ Ly6G^−^ Ly6C^−^ F4/80^+^ MHCII^+^), M1-like macrophages (CD45^+^ CD11b^+^ Ly6G^−^ Ly6C^−^ F4/80^+^ MHCII^+^ CD206^−^), M2-like macrophages (CD45^+^ CD11b^+^ Ly6G^−^ Ly6C^−^ F4/80^+^ MHCII^+^ CD206^+^), T cells (CD45^+^ CD3ε^+^), CD4^+^ T helper cells (CD45^+^ CD3ε^+^ CD4^+^), CD8^+^ cytotoxic T cells (CD45^+^ CD3ε^+^ CD8^+^), activated CD4^+^ T cells (CD45^+^ CD3ε^+^ CD4^+^ CD62L^+^ CD44^high^), activated CD8^+^ T cells (CD45^+^ CD3ε^+^ CD8^+^ CD62L^+^ CD44^high^) as described in^23^. The populations are expressed as a percent of parent. The parent populations are CD45^+^ CD11b^+^ Ly6G^+^ for mature granulocytes and G-MDSCs; CD45^+^ CD11b^+^ Ly6G^−^ for Mo-MDSC; CD45^+^ CD11b^+^ Ly6G^−^ Ly6C^−^ for TAM, total TAM for M1-like and M2-like macrophages, live single cells for CD45^+^ cells, CD45^+^ cells for CD3 cells, and CD3 T cells for CD4 and CD8 cells.

### BMDM polarization

Primary bone marrow was harvested from 6 to 8 weeks old FVB mice. The femurs and tibias were dissected, the epiphyses were removed and the bone marrow was flushed by centrifugation. Cells were cultured in Petri Dishes in DMEM/F12 with 10% FBS and M-CSF (20 ng/ml, Leinco Technologies) for 5 days. Cells were incubated for 3 days with filtered conditioned media from 4T1 cells. Briefly, the 4T1 cells were cultured to near confluency in DMEM with 10% FBS then fresh media was conditioned for 24 h by growing the 4T1 cells in DMEM without FBS. BMDM polarization was analyzed by flow cytometry using MHCII and CD206 antibody as described above. For the rescue experiments, anti Mouse-GM-CSF antibody (clone MP1-22E9, eBioscience, 1/500) was added to the conditioned media before incubation with BMDM.

### ELISA

Cells were seeded at 20% confluency in DMEM with 5% FBS and 2 ng/ml TGFβ and incubated for 3 days (each condition was performed in biological triplicate). Filtered supernatants (from equivalent number of cells) were analyzed by Eve Technologies using the multiplex bead platform Mouse Cytokine Array/Chemokine Array 31-Plex.

### Quantitative real-time RT-PCR (qRT-PCR)

Total RNA from 4T1-shScr and 4T1-sh*Snail1* cells was isolated from cells using the RNeasy Mini Kit (Qiagen). Complementary DNA (cDNA) was synthesized from 2 μg of total RNA using the Superscript First Strand kit (Invitrogen). mRNA levels were determined using semi quantitative reverse transcriptase using Fast SYBR Green (Applied Biosystems). Reactions were performed using the comparative threshold cycle (Ct) method for relative quantification. The glyceraldehyde-3-phosphate dehydrogenase (GAPDH) was used as an endogenous control. The data reported is the average of three technical replicates of two independent biological replicates. Primer sequences were designed using the PrimerBank website^45–47^ and are listed in Supplemental Table S1.

Total RNA was isolated from TAM (as defined in Flow Cytometry Analysis) sorted from 4T1-shScr and 4T1-sh*Snail1* tumors 14 days post transplant. Cells were processed and stained as described above and sorted on the ARIAII system (BD). RNA was isolated using the E.N.Z.A. Total RNA Kit (OMEGA) according to the manufacturer’s instructions. RNA was processed into cDNA using qScript cDNA SuperMix (Quantabio). Target genes were assessed using quantitative real-time PCR Taqman primer probes sets (Applied Biosystems). Relative gene expression was determined on an ABI7900HT quantitative PCR machine (ABI Biosystems) using Taqman Gene Expression Master Mix (Applied Biosystems). The threshold cycle method was used to determine fold change gene expression normalized to *Gapdh* and *tbp*.

### GM-CSF dosing in vivo

GM-CSF (100 ng per mouse) or saline solution were injected intraperitoneally every other day starting 7 days before tumor cells injections and for the duration of the experiment. After 7 days of GM-CSF injection, 10^6^ 4T1-shScr or 4T1-sh*Snail1* cells were injected in the right inguinal fat pad or 5.10^5^ 4T1-shScr or 4T1-sh*Snail1* were injected via the tail vein and the GM-CSF or saline solution injections were continued every other day for the remaining of the experiment. Mice were sacrificed 12 or 25 days after mammary transplants or 7 days after tail vein injection.

### Statistical analysis

Statistical analysis was performed using Prism 4 software (GraphPad Software). All data are presented as mean ± standard deviation. The two-tailed Student *t*-test was used. We considered *p* < 0.05 as significant.

## Electronic supplementary material

Supplementary Legends

Supplemetal Figure S1

Supplemental Figure S2

Supplemental Figure S3

Supplemental Table S1
