# Supplementary material for: SNAIL1 action in tumor cells influences macrophage polarization and metastasis in breast cancer through altered GM-CSF secretion
Source: Oncogenesis. 2018 Mar 29;7(3):32. doi: 10.1038/s41389-018-0042-x (PMC5874242; doi:10.1038/s41389-018-0042-x)
Supplement: Supplementary file 1 — Supplementary Legends [file 41389_2018_42_MOESM1_ESM.doc]

**Supplemental Figure S1. SNAIL1 deletion in mammary tumors and normal mammary glands**

(A) Total tumor burden of MMTV-PyMT, *Snail1*^fl/fl^ (WT, PyMT) and MMTV-PyMT, MMTV-Cre, *Snail1*^fl/fl^ (SNAIL1 KO, PyMT) mice, determined by the sum of the volume of all tumors for each mouse at 13 weeks of age. 14-15 mice per group. (B) Quantification of invasive and round tumors in MMTV-PyMT, *Snail1*^fl/fl^ (WT, PyMT) and MMTV-PyMT, MMTV-Cre, *Snail1* ^fl/fl^ (SNAIL1 KO, PyMT) mice when the largest tumor is 10 to 15 mm with representative tumors (C). scale bar, 1 cm. 8 to 11 mice per group. (D) Quantification of the distribution of hyperplasia/adenoma (blue), early carcinoma (pink) and late carcinoma (red) in MMTV-PyMT, *Snail1*^fl/fl^ (WT, PyMT) and MMTV-PyMT, MMTV-Cre, *Snail1*^fl/fl^ (SNAIL1 KO, PyMT) when the largest tumor is 10 to 15 mm with representative H&E staining images (E). scale bar, 100 μm. 6 to 10 tumors per group. (F) Mammary outgrowth of mammary gland #4 of *Snail1*^fl/fl^ (WT) and MMTV-Cre, *Snail1*^fl/fl^ (SNAIL1 KO) 4 week and 5 week old females. Outgrowth calculated as area with invading epithelium (dotted line) relative to the total area of the fat pad as shown in representative image (G, 5 week old). scale bar, 1 cm.

**Supplemental Figure S2: Characterization of Snail1 and Snail2 expression, lung metastasis size and T cell activation**

Western blot of extracts from MMTV-PyMT, *Snail1*^fl/fl^ (WT, PyMT) and MMTV-PyMT, MMTV-Cre, *Snail1*^fl/fl^ (SNAIL1 KO, PyMT) tumor cells (A) or 4T1-shScr and 4T1-sh*Snail1* cells (B) with anti-SNAIL1 or SNAIL2 antibodies. Size of lung metastasis measured from H&E staining represented as relative number of pixels of the area 21 days after orthotopic transplants of 4T1-shScr (shScr) or 4T1-sh*Snail1* (sh*Snail1*) (10 mice per group) (C) or MMTV-PyMT, *Snail1*^fl/fl^ (WT, PyMT) and MMTV-PyMT, MMTV-Cre, *Snail1*^fl/fl^ (SNAIL1 KO, PyMT) mice when the largest primary tumor is 10 to 15 mm (5 to 8 mice) (D). Quantification of tumor infiltrated activated CD4 T cells (CD45^+^ CD3ε^+^ CD4^+^ CD62L^+^ CD44^high^) (E) or activated CD8 T cells (CD45^+^ CD3ε^+^ CD8^+^ CD62L^+^ CD44^high^) (F) in 4T1-shScr (shScr) and 4T1-sh*Snail1* (sh*Snail1*) mice 12 days post transplant expressed as a percent of total CD4^+^ T cells (E) or as a percent of total CD8^+^ T cells (F). 5 mice per group.

**Supplemental Figure S3: SNAIL1 modulates macrophage polarization in an MMTV-PyMT derived cell line and characterization of immune infiltrates in mice treated with GM-CSF**

(A) In vitro M1-like polarization of bone marrow derived macrophages (BMDM) by conditioned media from PMT-shScr or PMT-sh*Snail1* expressed as a percent of total BMDM. Representative results of 2 biological replicates with 3 technical replicates. (B to E) Immunophenotyping of tumors from 4T1-shScr (shScr) transplants and 4T1-shScr with injections of GM-CSF (shScr+GM-CSF) 12 days (B and D) and 25 days (C and E) post transplant. Myeloid populations (B and C) and T cells (D and E) as defined in Figure 2. 4 to 5 mice per group, *p<0.05 by unpaired t-test.

**Supplemental Table S1. qRT-PCR primers**
