# Supplementary figures and images for: SNAIL1 action in tumor cells influences macrophage polarization and metastasis in breast cancer through altered GM-CSF secretion
Source: Oncogenesis. 2018 Mar 29;7(3):32. doi: 10.1038/s41389-018-0042-x (PMC5874242; doi:10.1038/s41389-018-0042-x)

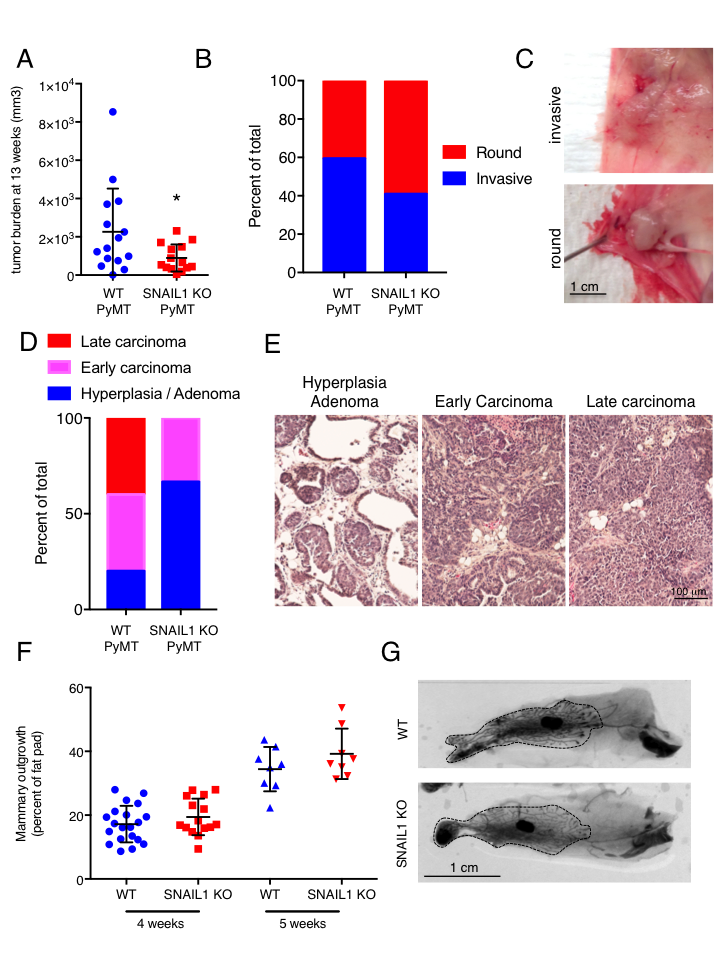

Supplement: Supplementary file 2 — Supplemetal Figure S1 [file 41389_2018_42_MOESM2_ESM.tif]

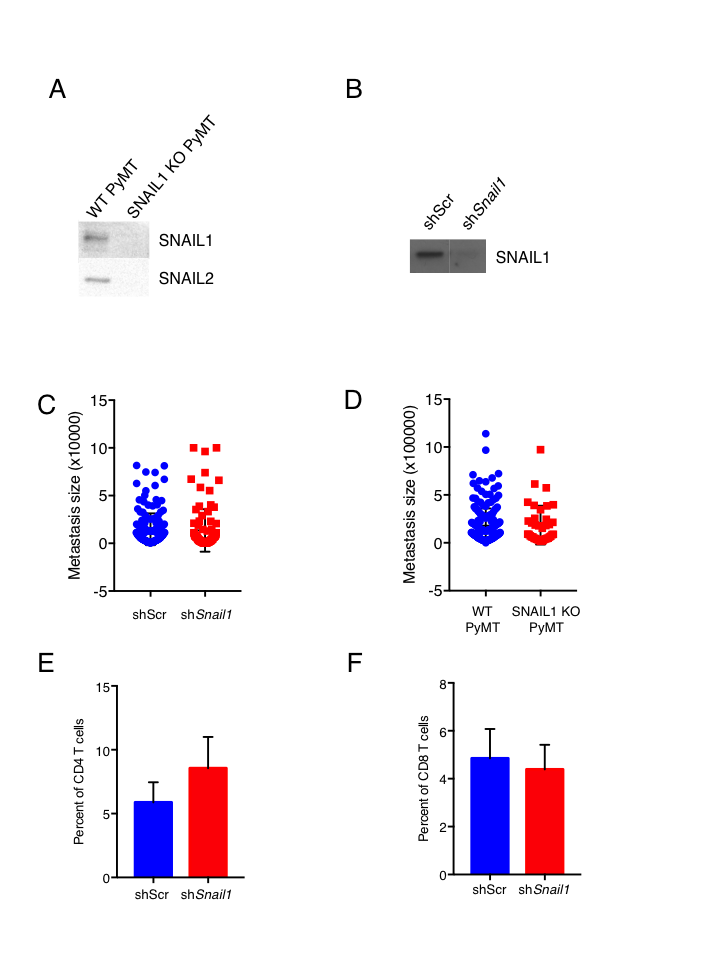

Supplement: Supplementary file 3 — Supplemental Figure S2 [file 41389_2018_42_MOESM3_ESM.tif]

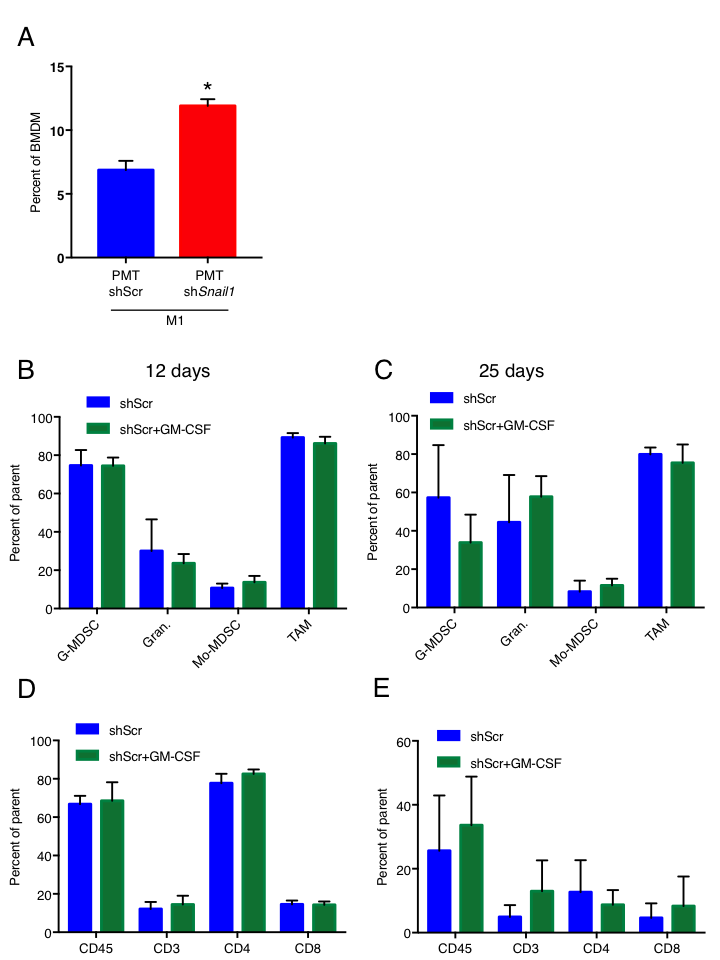

Supplement: Supplementary file 4 — Supplemental Figure S3 [file 41389_2018_42_MOESM4_ESM.tif]

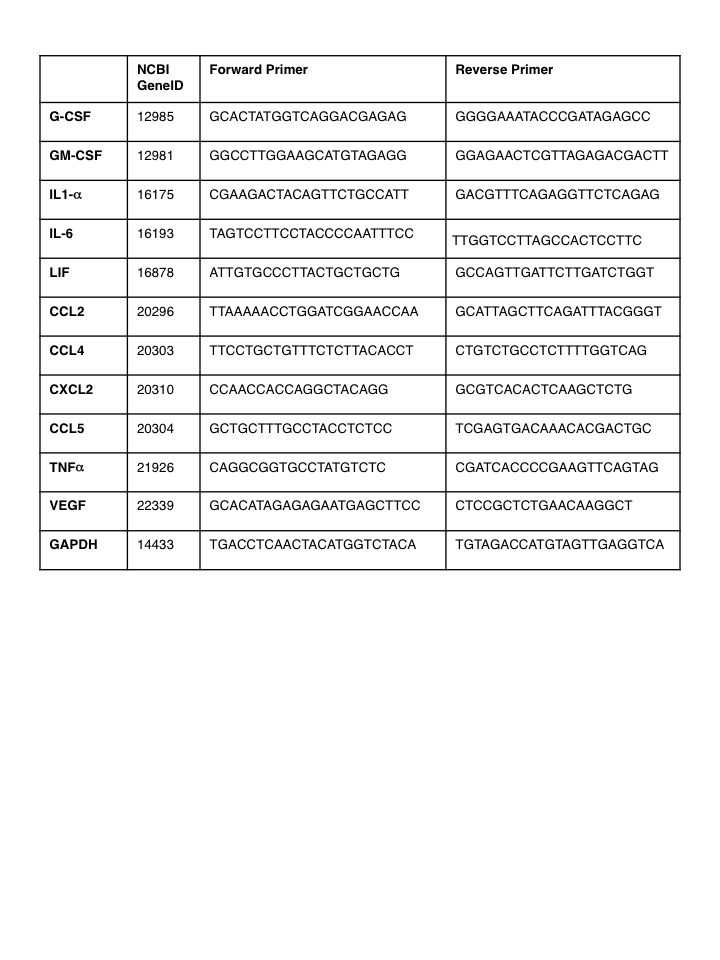

Supplement: Supplementary file 5 — Supplemental Table S1 [file 41389_2018_42_MOESM5_ESM.tif]
